# Supplementary material for: Alterations in Intratumoral Immune Response before and during Early-On Nivolumab Treatment for Unresectable Advanced or Recurrent Gastric Cancer
Source: Int J Mol Sci. 2023 Nov 22;24(23):16602. doi: 10.3390/ijms242316602 (PMC10706573; doi:10.3390/ijms242316602)
Supplement: Supplementary file 1 [file ijms-24-16602-s001.zip › Supplementary Figures_YS.pdf]

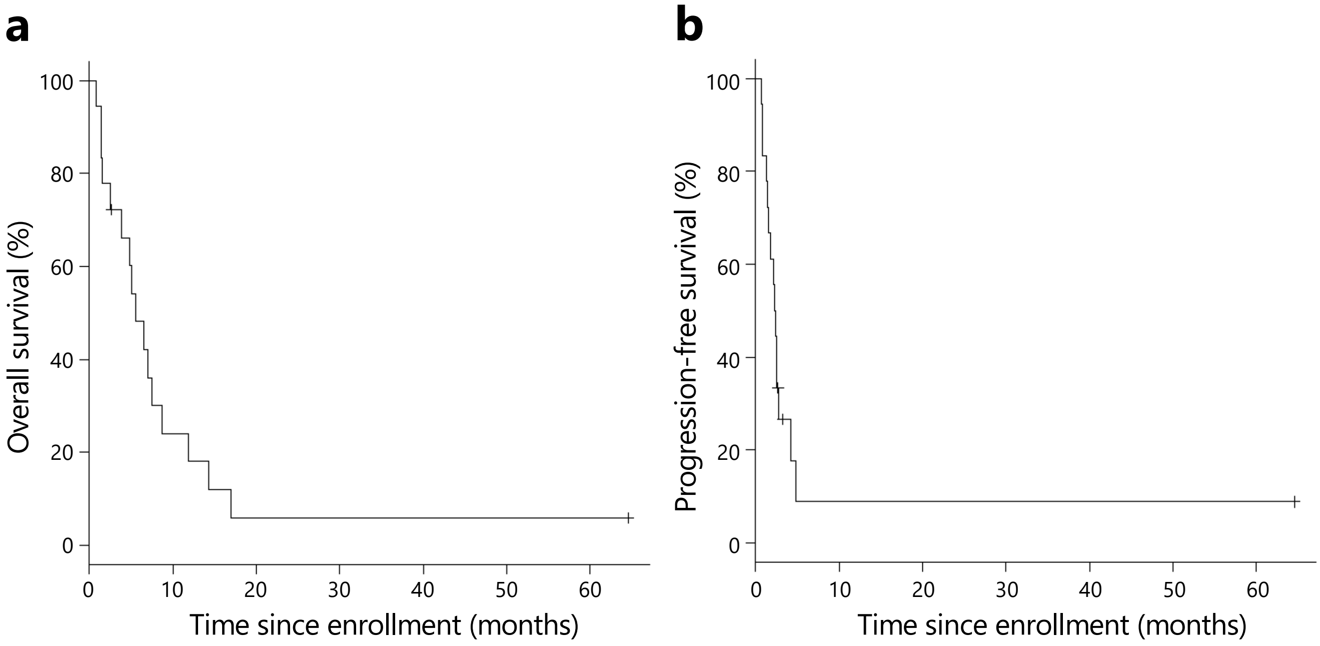

Figure S1. The Kaplan–Meier survival analysis. Survival curves of overall survival (a) and progression-free survival (b) of patients for immunological analysis (n=18).

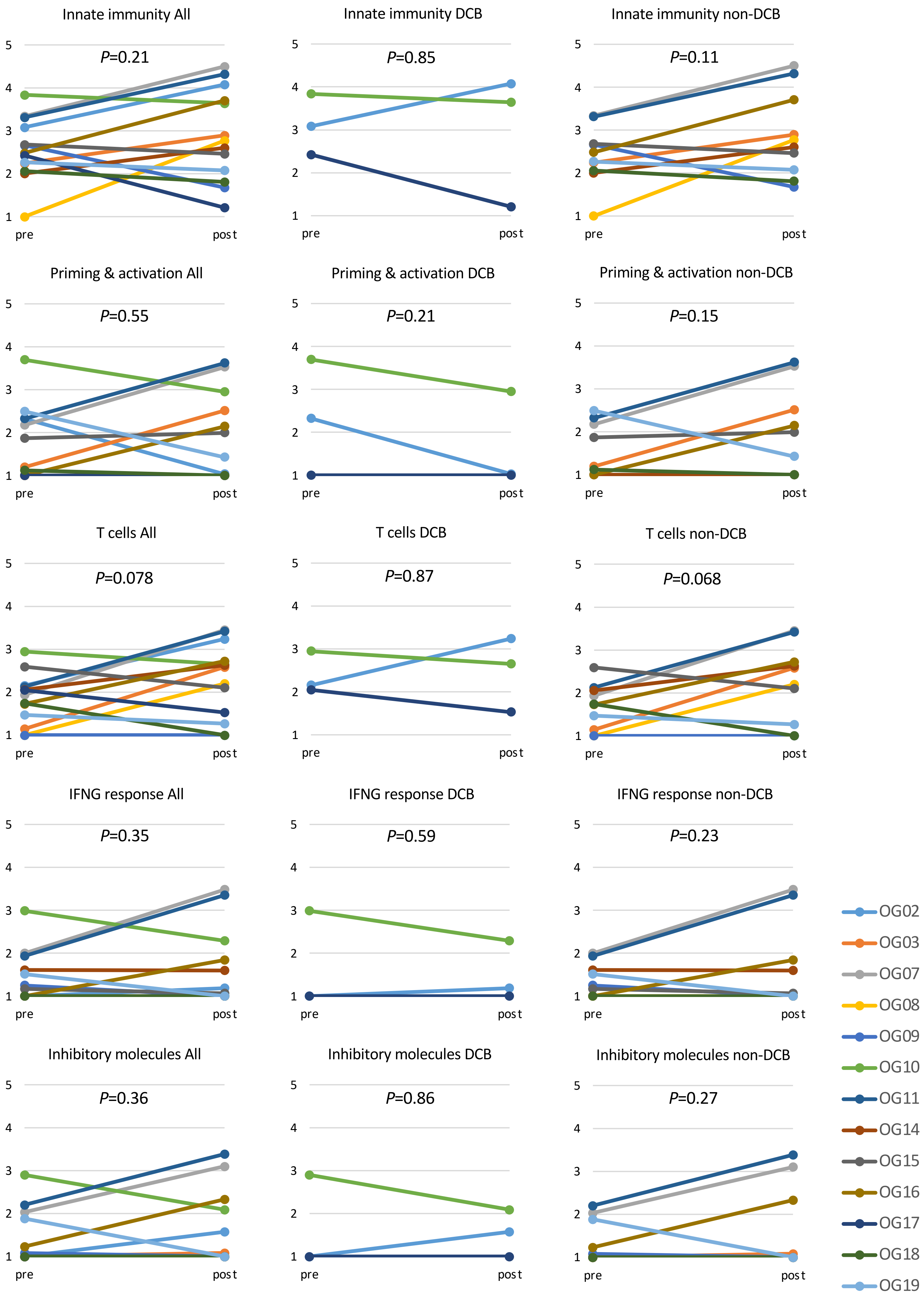

Figure S2-1. The comparison of immunogram scores between pre-treatment (pre) and early-on-treatment (post) in immunological cases (All), durable clinical benefit (DCB) and non-DCB patients.

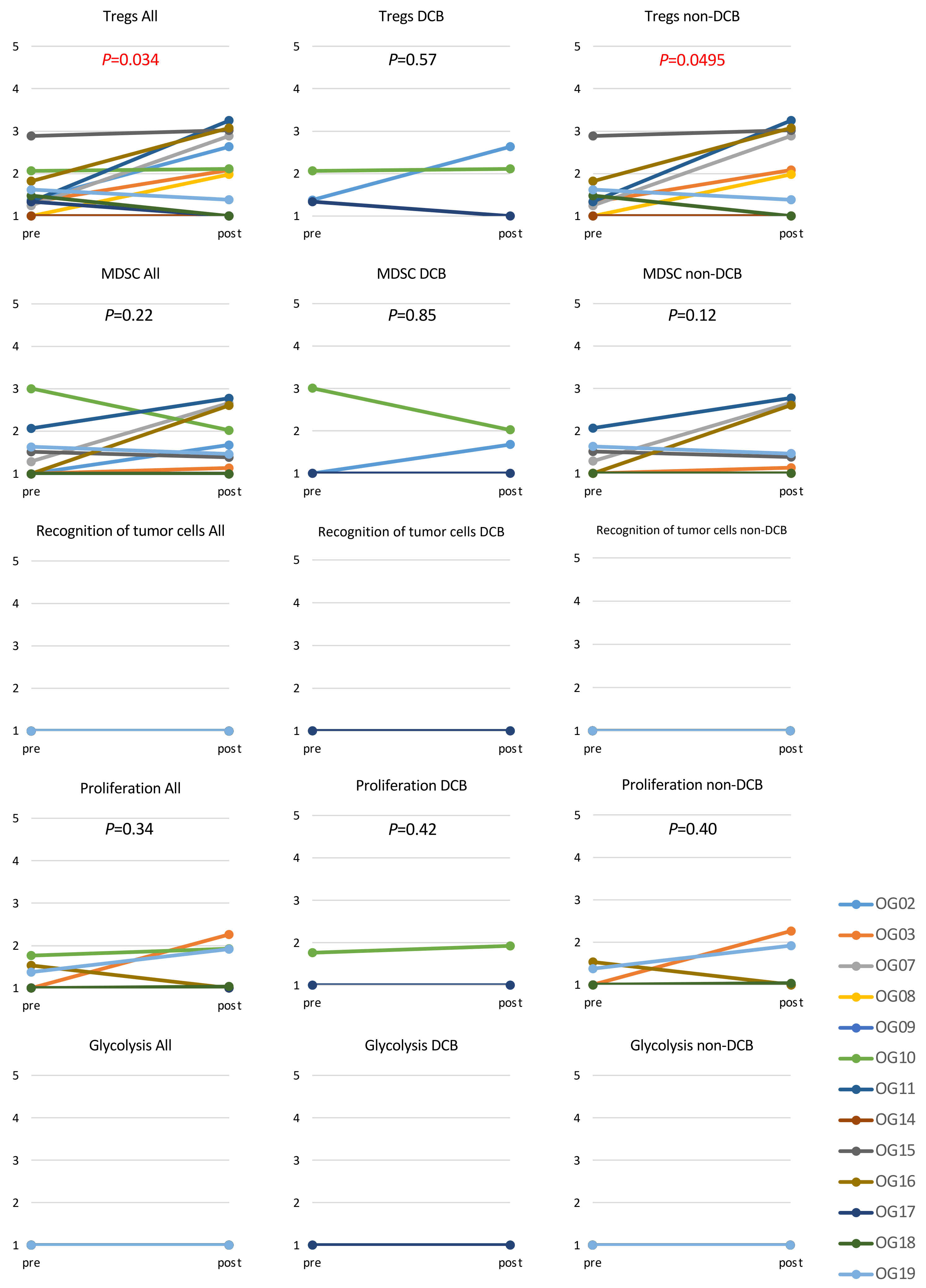

Figure S2-2. The comparison of immunogram scores between pre-treatment (pre) and early-on-treatment (post) in immunological cases (All), durable clinical benefit (DCB) and non-DCB patients.

pre-treatment

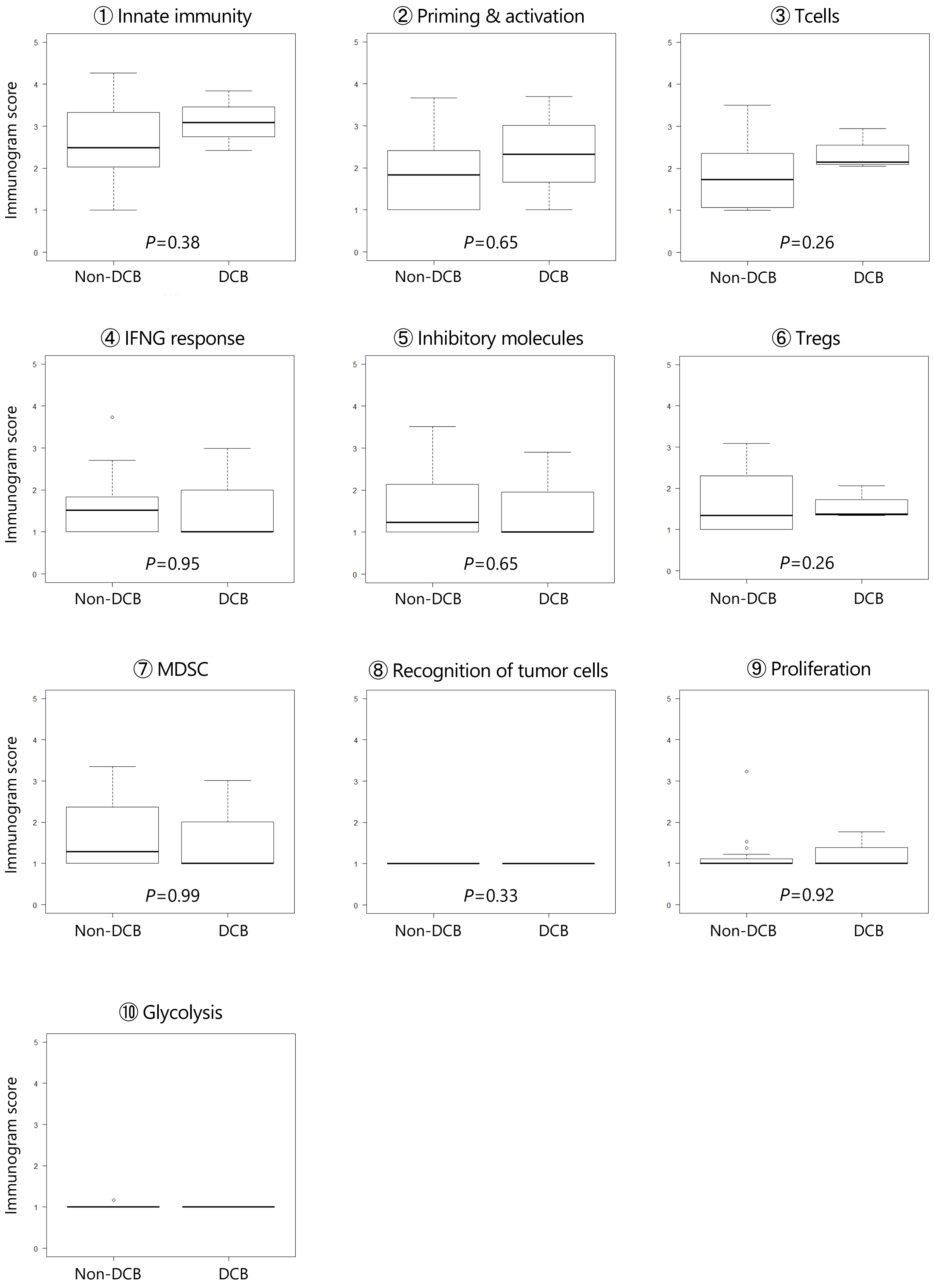

Figure S3. The comparison of immunogram scores of pre-treatment tumors between durable clinical benefit (DCB) and non-DCB patients.

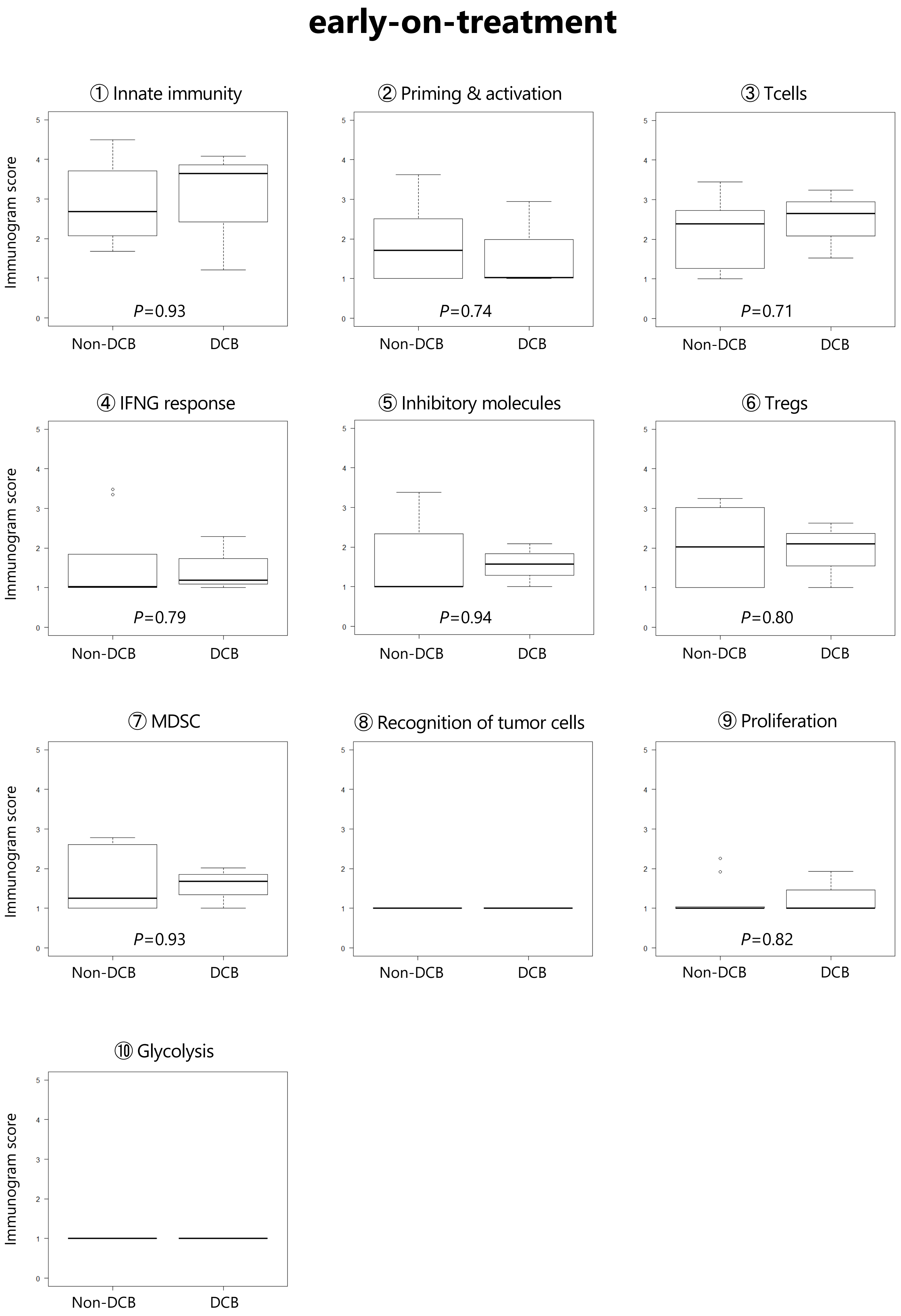

Figure S4. The comparison of immunogram scores of early-on-treatment tumors between durable clinical benefit (DCB) and non-DCB patients.

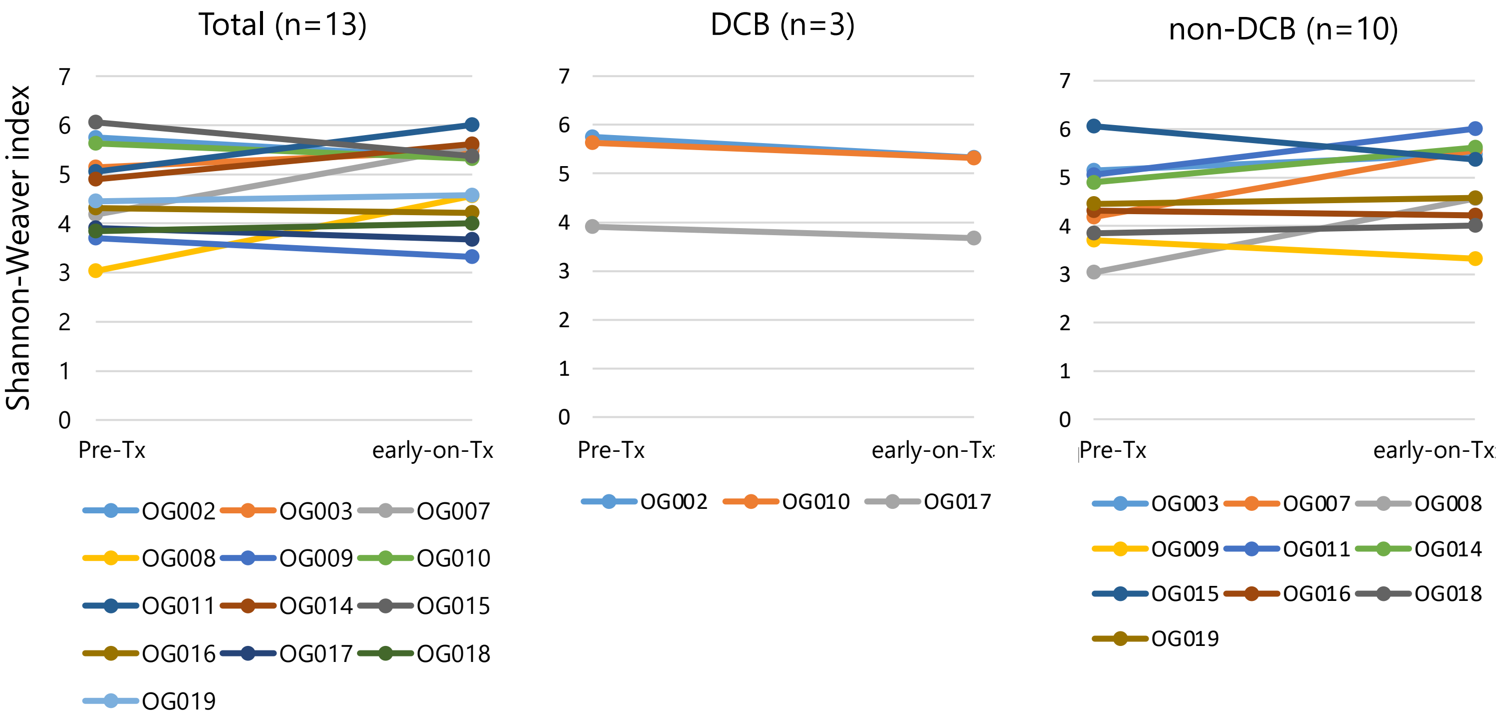

Figure S5. Comparison of TCRβ repertoire diversity between pre-treatment (Pre-Tx) and early-on-treatment (early-on-Tx) tumors. The Shannon-Weaver indices were used to compare the diversity of TCRβ repertoires in 13 patients for whom pre-treatment and early-on-treatment paired samples were available.
